# High Hardness and Wear Resistance in AlCrFeNiV High-Entropy Alloy Induced by Dual-Phase Body-Centered Cubic Coupling Effects

**DOI:** 10.3390/ma15196896

**Published:** 2022-10-05

**Authors:** Cong Feng, Xueliang Wang, Li Yang, Yongli Guo, Yaping Wang

**Affiliations:** 1 MOE Key Laboratory for Nonequilibrium Synthesis and Modulation of Condensed Matter, School of Physics, Xi’an Jiaotong University, Xi’an 710049, China; 2 MOE Key Laboratory of Thermo-Fluid Science and Engineering, School of Energy and Power Engineering, Xi’an Jiaotong University, Xi’an 710049, China

**Keywords:** high-entropy alloy, multiphase BCC, hardness, wear resistance

## Abstract

High-entropy alloys (HEAs) with high hardness are promising materials for advanced industrial manufacturing. In this study, the AlCrFeNiV HEA was designed and successfully prepared using a plasma instantaneous process. The hardness test showed that AlCrFeNiV had a high hardness of 1076 ± 15 HV, which was much higher than those reported in the literature. The microstructure of AlCrFeNiV was composed of two different types of body-centered cubic (BCC) structures, BCC1 (Al, Cr, Fe, and Ni) and BCC2 (enriched V and Cr). A mixture of different BCC systems produced solution strengthening, which was responsible for the superior hardness. Moreover, the reciprocating sliding wear behavior of HEA against Al_2_O_3_ balls under dry and lubricated conditions at ambient temperature was investigated. The wear rates of AlCrFeNiV against Al_2_O_3_ under dry wear and lubrication were 17.2 × 10^−5^ mm^3^ N^−1^·m^−1^ and 12.4 × 10^−5^ mm^3^N^−1^·m^−1^, respectively, which were of the same order of magnitude as the wear rates of BCC HEAs. Regardless of the dry wear or wear with lubrication, the wear mechanism of the HEA was abrasive and delamination wear.

## 1. Introduction

High-entropy alloy (HEA) has been favored by many researchers because of its excellent properties, such as its superb specific strength [1], good fatigue behavior [2], wear and corrosion resistance [3,4,5], super-conductivity [6], and radiation tolerance [7], etc. Due to these attractive properties, HEAs are promising candidates for technological applications in many fields, such as high-temperature applications [8], nuclear energy industry [9], space exploration, and storage of cryogens [10]. HEA was focused on high hardness for advanced industrial manufacturing. It is generally believed that materials with high hardness will have less slip and fewer dislocation vulnerabilities to resist cutting by force. Among these properties, wear is critical to the service lifetime of metallic parts with relatively moving contact surfaces, such as bearings and gears. Friction and wear during service may cause local deformation and failure of materials [11,12]. Therefore, developing wear-resistant metal alloys has been in high demand. Compared with traditional commercial alloys, HEA has excellent hardness, oxidation resistance, softening resistance and other properties, showing excellent wear resistance [13,14,15,16,17], opening up a new way for the development of industrial alloys to explore new wear-resistant materials.

HEAs, which were first independently and respectively reported by Yeh et al. [18] and Cantor et al. [19], showed great application potential since 2004. Different from the traditional strategy, HEAs are not based on only one or two principal elements but generally contain at least five principal elements in equimolar or near-equimolar proportions [20]. Each principal element should have a concentration range from 5 to 35 at.% [19,21]. The equiatomic or near-equiatomic HEA design aims at the maximization of the configurational entropy to stabilize the solid solution phase. HEAs are often treated as multi-component, solid-solution alloys with simple crystal structures, such as the face-center-cubic (FCC) phase [1,2,3], body-center-cubic (BCC) phase [22], hexagonal-close-packed (HCP) phase [22,23] or their mixtures.

To improve the wear resistance of HEAs, many studies have been made on the phase composition and microstructure of HEAs. In HEA systems, the choice of a HEA principal element will lead to the change of the alloy structure and then lead to the change of the alloy properties. Wang et al. [24] reported that with the increase in Al content, the structure of FeCrNiMnAl_x_ was transferred from FCC to BCC+B2 and the average friction coefficient and wear rate of the FeCrNiMnAl_x_ alloys were reduced obviously until x = 0.8. Moazzen et al. [25] found that in the Fe_x_CoCrNi HEA alloy system, the volume of BCC increases obviously, resulting in the improvement of hardness wear resistance by increasing the amount of Fe from 0 to 1.6, accordingly. Treutler et al. [26] found that Cr_27.5_Co_14_Fe_22_Mo_22_Ni_11.65_V_2.8_ shows the potential of generating a remelted wear- resistant alloy. Nussbaum et al. [27] prepared (AlCrTiV)N by cathodic arc evaporation and found that with the addition of a N element, the (AlCrTiV)N HEA coating consists of TiN ceramics as well as a few Laves phases, which significantly improved the hardness and tribological performance of the coatings. Aluminum and chromium elements are widely used in those compositions for their effects on microstructure, strengthening and oxidation resistance of alloys [28]. Vanadium is often used to improve the frictional and wear properties because of its atomic size deforming the crystal structure of both BCC and FCC alloys [29]. Since the atomic radii of Al and V are much larger than those of other alloying elements, the addition of those elements will cause the atomic level stress of the HEA. In contrast, the BCC phase has a looser structure than the FCC phase. With the increase in Al content, the atomic stress of the HEA increases, which promotes the transformation of FCC structure to BCC structure, thus reducing the strain energy and Gibbs free energy [30].

In this work, a new strategy is designed to solve the aforesaid problems. The high purity and densification of HEAmaterials can be hence obtained by continuous casting. In addition, the friction and wear tests were carried out on AlCrFeNiV HEA against Al_2_O_3_ under dry wear and lubrication wear. The AlCrFeNiV alloy produced in this work is expected to exhibit improved mechanical performance that can be applied in cutting tools.

## 2. Materials and Methods

### 2.1. Preparation of AlCrFeNiV

It has been found that most of the high entropy alloys consisting of solid solution phase do not strictly meet the requirements limitations proposed by the Hume-Rothery criterion. Therefore, based on the criterion, the present research proposed a series of semi-empirical physical parameters that predict the composition of the alloy phase and assist in alloy design. These parameters mainly include mixing entropy (Δ*S_mix_*), mixing enthalpy (Δ*H_mix_*), atomic radius difference (*δ*), valence electron concentration (*VEC*) and electronegativity difference (Δ*χ*) [31] and parameter Ω [32,33], which is a comprehensive reflection mixing entropy of mixing the common effect, etc. Table 1 gives all the above parameters of AlCrFeNiV. It can be seen from the experiment summarized in the research that the formation conditions of multi-phase BCC are *δ* ≤ 7.1%, −22.9 KJ·mol^−1^ ≤ Δ*H_mix_* ≤ 0.3 KJ·mol^−1^, *VEC* ≤ 7.5, Δ*χ* ≤ 16.6%.

The five alloying elements selected in this work are properly within this range. The results of this work contribute to the verification and refinement of the empirical formulas for predicting the phase structure of HEAs.

Equiatomic AlCrFeNiV HEA was synthesized by mechanical alloying and then by a plasma sintering process. Specifically, Al, Cr, Fe, Ni and V powders with purity greater than 99.5% and an initial particle size of 75 μm were selected as raw materials. This was followed by high-energy ball milling (ball to powder ratio is 10:1) in a SPEX 8000 mill for 2 h under argon protection at room temperature. The raw materials were prepared in a shock ball milling followed by continuous casting. Continuous casting is a method to obtain a HEA sheet with high purity and density by using the grain boundary discharge of mixed powder under plasma, which is assisted by high pressure to melt the grain boundary of metal powder, and then extruding the liquid flow under the action of high-pressure extrusion. The progress of SPS was conducted at 1100 °C for 1 h with a constant axial pressure of 40 MPa. Then, the AlCrFeNiV HEA was cooled down to room temperature by furnace cooling.

### 2.2. Microstructure Characterization

Structural characterization was conducted using X-ray diffractometry (XRD), scanning electron microscopy (SEM) and transmission electron microscopy (TEM). BRUKER D8 ADVANCE XRD equipment was used to evaluate the phases present, crystal structures and the lattice constants. The incident beam used in XRD was Cu Kα radiation (wavelength 0.154 nm), with the scans performed between a 2θ of 20°~100° with a step size of 12° min^−1^. Zeiss Gemini SEM 500 was coupled with energy-dispersive X-ray spectroscopy (EDX); the accelerating voltage of SEM was 10 kV and the beam current was 20 pA. When converted to EDX, the accelerating voltage was increased to 15 kV. JEOL 2100 TEM with the voltage 120 kV was used for further structural characterization. The microstructure was analyzed at several length scales, first using SU3500 SEM equipped with the electron backscattered diffraction (EBSD) detector from Oxford Instruments and controlled by the Oxford AZtec software. High-resolution EBSD maps were collected at an accelerating voltage of 20 kV using a spot size of 160 nm.

### 2.3. Mechanical Tests

To evaluate the mechanical performance of HEA, hardness was evaluated with loading of 5 N and a corresponding holding time of 15 s. Each Vickers hardness value was obtained by averaging 10 indents tests on a mirror-like surface. To explore the wear resistance of HEA, the tribological behavior of HEA sliding under dry conditions and lubrication was evaluated by using a ball-on-disk tribometer (HT-1000), selecting Al_2_O_3_ balls as the grinding material. The test was carried out at a normal load of 10 N with a sliding speed of 0.18 m s^−1^ and a duration of 30 min. Each wear test was repeated at least three times to ensure reliability. The friction coefficient was recorded in real time by software. The wear rates of disc and ball were calculated by

(1) $$W=\frac{\Delta m}{\rho \cdot F\cdot L}$$

where Δ*m*, *ρ*, *F* and *L* are the wear weight loss, density normal load and total sliding distance, respectively. The density of samples was measured by Archimedes’ drainage method (ZMD-2). The weight loss was derived from the quality difference of the patterns before and after the friction test, and each mass was weighed more than five times to reduce the error.

## 3. Results and Discussion

**Figure 1:**
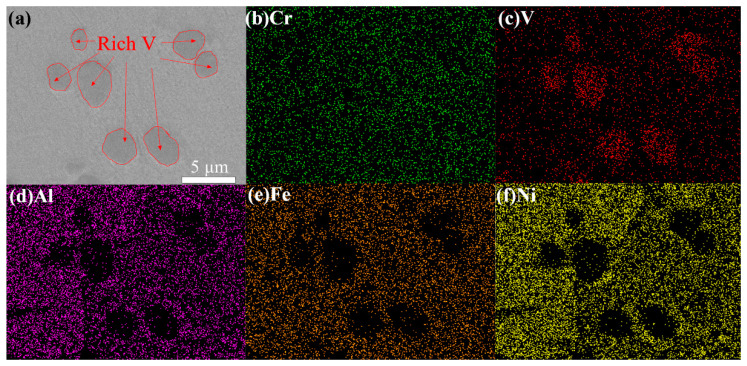
(**a**) SEM image of AlCrFeNiV and corresponding EDX mapping showing spatial distribution of (**b**) Cr, (**c**) V, (**d**) Al, (**e**) Fe, (**f**) Ni of the investigated area.

**Figure 2:**
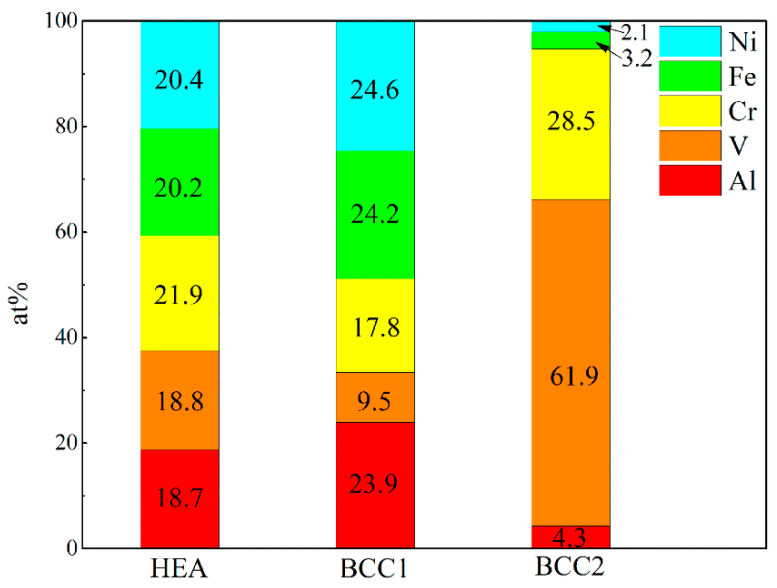
Columnar stacking diagram (at.%) of HEA, BCC1 and BCC2.

The microstructure morphology and element dispersion in the AlCrFeNiV alloy synthesized by plasma were investigated and the SEM morphology and corresponding EDX are shown in Figure 1. Specifically, as shown in Figure 1a, regions with different gray colors were displayed in the SEM image. Figure 1b–f show the corresponding EDX mapping. It indicates that regions with the dark gray color in Figure 1a correspond to vanadium (V) aggregations (Figure 1c); the rest of the metal elements, such as Al, Cr, Fe, and Ni, are uniformly dispersed in the alloys. Figure 2 shows the columnar stacking diagram (at.%) of HEA, BCC1 and BCC2.

Figure 2 shows HEA and the columnar accumulation diagram (at.%) of element content in the two phases. The total distribution of elements is not much different from that of a theoretical Moore atom. It can be seen from Figure 1 that BCC1 lacks a V element and its content is obviously low. In contrast, BCC2 contains 61.9 at.% V. The contents of other elements, except Cr and V, are all lower than 5 at.%. This is consistent with the surface scanning results in Figure 1.

**Figure 3:**
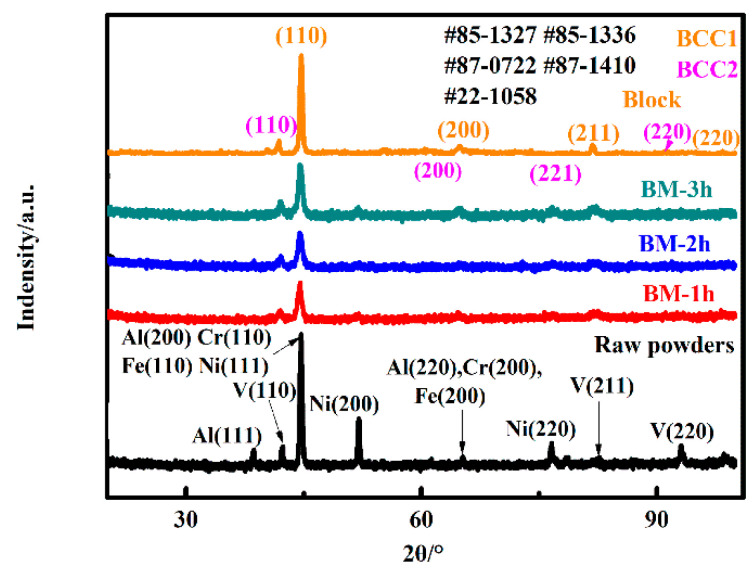
XRD patterns of the AlCrFeNiV block and powders with different ball milling times.

XRD patterns were used to study the effect of ball milling on the powder structure of AlCrFeNiV alloy, as shown in Figure 3. It is obvious that the peaks of the five main elements in the original mixed powder are clearly visible. With the increase in milling time, the intensity of Al(200), Cr(110), Fe(110) and Ni(111) peaks began to decrease, and even the peaks of Al(111), Ni(200), Ni(220) and V(220) disappeared, which may be caused by solid solution in the process of high-energy milling.

**Figure 4:**
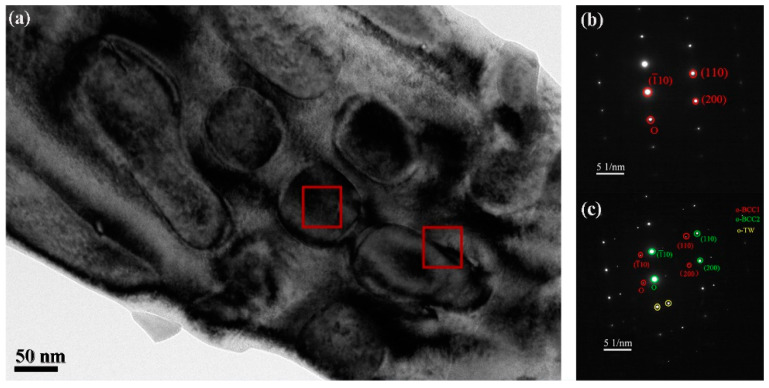
(**a**) corresponding TEM images of the AlCrFeNiV high-entropy alloy with (**b**,**c**) different electron diffraction patterns.

Figure 4a shows the TEM image of AlCrFeNiV high-entropy alloy, and Figure 4b,c show the corresponding electron diffraction patterns. Figure 4b,c show that the AlCrFeNiV alloy is not just a single body-centered cubic (BCC), but a mixture of two BCC structures with different lattice constants. It can be seen from the XRD patterns that the powder evolution process of BCC2 is dominated by V element. Moreover, in Figure 4c, there are very few twins in the electron diffraction.

**Figure 5:**
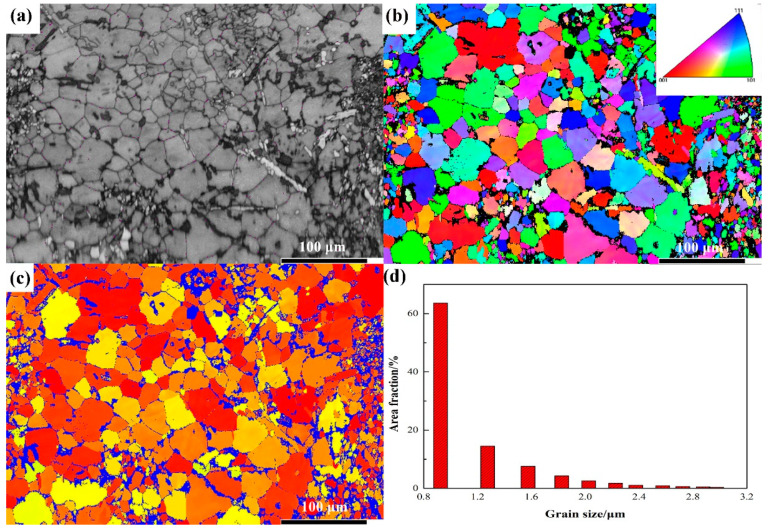
(**a**) Band contrast (BC) map superimposed by twin boundaries (purple lines) of AlCrFeNiV. (**b**) corresponding EBSD IPF subset maps. (**c**) corresponding Taylor Factor maps and (**d**) histogram of grain size distribution.

Figure 5 shows the EBSD results of the AlCrFeNiV. Figure 5a shows that the band contrast (BC) map is superimposed by purple twin boundaries of AlCrFeNiV, which shows the general microstructure. Figure 5b presents a large-scale EBSD Inverse Pole Figure (IPF) map of the AlCrFeNiV sample. Figure 5c shows Taylor Factor maps of AlCrFeNiV, which shows the ease of crystal slip. Taylor Factor [34] can be defined as:

(2) $$M=\frac{\sigma_{x}}{\tau }=\frac{d\gamma }{d\varepsilon_{x}}$$

where *σ_x_* is normal stress in polycrystals; *ε_x_* is normal strain; *τ* is shear stress and *γ* is shear strain. The red areas (in Figure 5c) are the areas with high Taylor Factor values, indicating that the deformation of these grains is difficult and requires high deformation energy. Figure 5d is the corresponding histogram of grain size distribution, which indicates that the average grain size is 450 nm.

To evaluate the effect of microstructure evolutions on the mechanical performance of the AlCrFeNiV HEA, the microhardness was tested and further compared with the value reported in the literature. The hardness of the AlCrFeNiV alloys reached 1076 ± 15 HV0.5. From the above TEM and EBSD results, the microstructure characteristics, such as grain size and dislocation density of AlCrFeNiV, are practically identical at submicron scale. The superhigh hardness obtained in the AlCrFeNiV HEA can be attributed to the two different BCC crystal structures formed by the high-energy ball milling combined with a plasma process [32]. 

**Figure 6:**
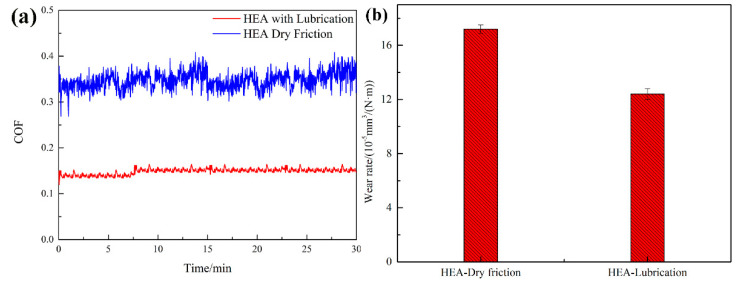
(**a**) Typical friction coefficient curves of the HEA dry wear and HEA with lubrication. (**b**) Wear rate of the HEA dry wear and HEA with lubrication sliding against Al_2_O_3_.

To further investigate the mechanical properties of HEA, friction and wear tests were carried out on HEA. Figure 6 shows the typical RT coefficient of friction (COF) curves of the HEA dry friction and HEA with lubrication and their corresponding wear rate against Al_2_O_3_. As can be seen from Figure 6a, the friction coefficient of HEA with lubricant added fluctuates more gently in the whole sliding process, and the friction coefficient is also smaller. The average coefficient of friction of HEA with lubrication is only 0.15, whereas the COF of HEA dry friction can reach 0.34, which is more than twice that of the COF with lubrication. Even in the state of dry friction, the friction coefficient of HEA is at a relatively low level. It is related to the fact that dual-phase BCC significantly improves the hardness of the metal, thus improving the impervious ability of the metal and significantly reducing the surface ploughing. The decrease in the COF was more obvious under the lubrication of lubricant. After about 450 s, the friction coefficient of HEA with lubrication tends to be stable with minimal fluctuation. This may be due to the lubrication and cooling of the worn surface by the resin, reducing the accumulation of hard abrasive particles during the wear process. As a result, the friction heat generated by the worn surface can be quickly transferred to the surrounding area. Thus, the friction coefficient curve is kept at a relatively stable level. Comparatively, the friction coefficient fluctuates greatly under dry wear due to the continuous cycle of oxide film formation and tearing on the surface.

The wear rate is an important parameter to judge the wear resistance of materials. The wear rate of HEAs in the dry and lubrication conditions is shown in Figure 6b. To be more exact, the wear rate of the dry wear is higher than that of lubrication. The wear rates of the HEA in dry and lubrication conditions were approximately 17.2 × 10^−5^ mm^3^ N^−1^ m^−1^ and 12.4 × 10^−5^ mm^3^ N^−1^ m^−1^, respectively. According to a report by Haghdadi et al. [35], the hardness and plasticity of the alloy under grinding conditions jointly determine material loss under medium and low load conditions. Although the hardness of AlCrFeNiV with a dual-phase BCC structure is very high, its plasticity is poor. When it is worn by the grinding ball with higher hardness, the material surface is prone to fracture, which means the increase in crushing degree, which is often accompanied by the generation of many fragments on the contact interface. They have sharp edges and a secondary grinding effect during sliding, increasing wear rates. In addition, the wear rate of the alloy in deionized water is significantly lower than that in dry conditions, probably due to lubrication.

**Figure 7:**
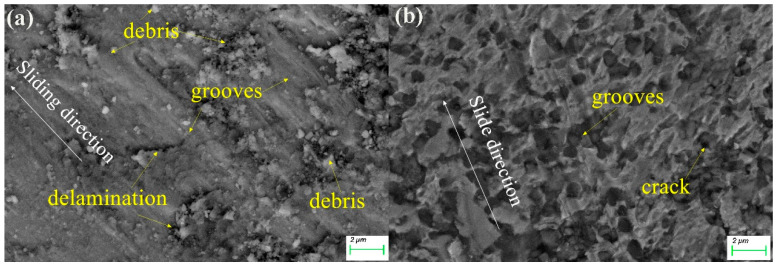
The worn surface SEM image of (**a**) the HEA dry wear and (**b**) HEA with lubrication.

Figure 7 shows the morphology of the worn surface of the alloys under dry conditions and HEA with lubrication. It is manifest from the Figure 7 that there is a significant difference on the worn surface between HEA dry wear and HEA with lubrication. The surface delamination phenomenon is obvious, which is due to the relatively hard Al_2_O_3_ sphere pressed into the soft alloy surface, resulting in serious deformation and grooves. In addition, many broken wear debris particles were found attached to the surface, indicating that the surface suffered from severe adhesive wear. A great deal of thin and dense grooves parallel to the sliding direction can be found, which is typical of abrasive wear. In the case of adding lubricant, the number of grooves is significantly reduced, and the number of abrasive chips is sharply reduced. Apparently, abrasive wear occurred during the sliding process. Moreover, some delamination and deformed areas were observed. Therefore, the wear mechanism of HEA dry friction is mainly abrasive wear and stratified wear. It is noteworthy that there are a few delamination areas and randomly distributed grooves on the wear surface caused by chip sliding and rolling, indicating mixed wear of two-body abrasives. The wear debris becomes fine particles, indicating the possibility of oxidation on the worn surface. As shown in Figure 7b, the wear surface of HEA with lubrication is smoother and the grooves become shallower.

**Figure 8:**
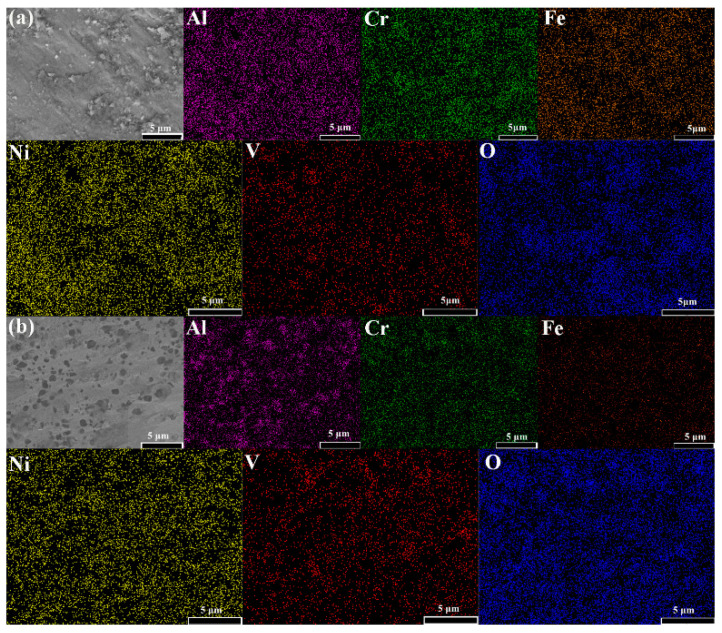
The worn surface SEM image and corresponding EDX mapping of (**a**) HEA dry wear and (**b**) HEA with lubrication.

**Figure 9:**
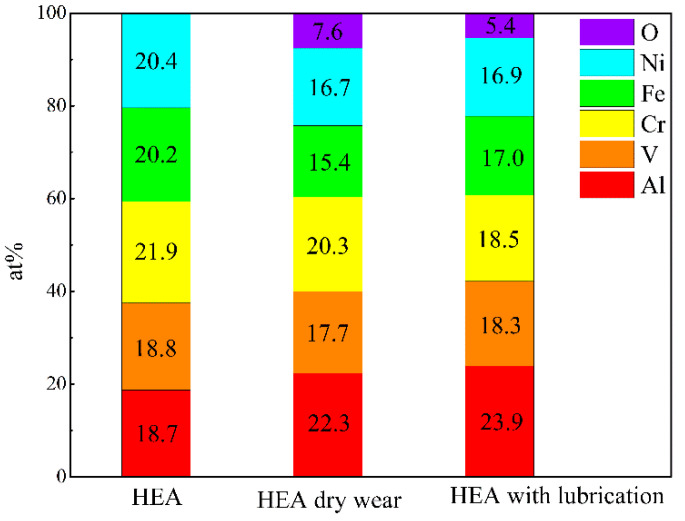
Columnar stacking diagram (at.%) of HEA and after wear.

The EDX mappings of the worn surface are shown in Figure 8. As can be seen from the figure, oxygen elements are evenly distributed on the wear surfaces of HEA and HEA with lubrication regardless of the presence of lubricants. The friction heat generated during the friction process results in oxidation of the surface. Therefore, the wear mechanism is mixed abrasive wear, and the wear medium is the oxide layer. Figure 9 shows the columnar stacking diagram (at.%) of elements distribution of HEA with dry wear and lubricant wear. It can be seen from the results that the theoretical values of each element content are close to the experimental values before the wear test. However, after sliding, the content of each element began to decline; but compared with almost no change, the oxygen element emerged as a new element. However, where the oxygen element is concerned, it is found that the oxygen content varies with the friction conditions. The oxygen content of dry friction is higher than that of friction with lubricant. Even so, the difference in oxygen content is not very large, only 2.2 at.%.

The energy consumed by the friction system mainly exists in the form of wear, material deformation and friction heat [36,37]. Therefore, the influence of load on wear performance is analyzed from the following several aspects. First, in the process of dry sliding contact, the larger applied load will produce higher contact stress and shear stress. As a result, contact surface damage is more severe, resulting in increased wear rates. The maximum Hertz contact stress *σ_max_* of planar and spherical contact can be calculated as follows [35,36,37], taking alumina as an example:

(3) $$\sigma_{max}=\frac{3P}{2\pi a}$$

(4) $$a=\sqrt[{3}]{\frac{3PR}{4E'}}$$

(5) $$\frac{1}{E^{\prime }}=\frac{1-\upsilon_{1}^{2}}{E_{1}^{'}}+\frac{1-\upsilon_{2}^{2}}{E_{2}^{'}}$$

where *R* = 2.5 mm is the radius of Al_2_O_3_ ball, *E*_1_ = 340 GPa and *E*_2_ = 228 GPa represent the elastic modulus of the Al_2_O_3_ ball and AlCrFeNiV HEA disk, respectively, *υ*_1_ = 0.22 is the Poisson’s ratio of the alumina ball and *υ*_2_ = 0.3 is chosen for the HEA disk. When the normal load *P* is 10 N, the corresponding Hertzian contact stress of HEA was calculated to be 1887 MPa. All the Hertz contact stresses exceed the yield strength of the BCC1 matrix (~4800 MPa) but are much lower than the yield strength of BCC2 (~6100 MPa). Therefore, it can be reasonably inferred that the BCC matrix bears plastic deformation, whereas the hard BCC2 phase particles hinder the overall grain boundary movement and plastic flow. Due to the presence of a high density of BCC2, the HEA can endure such high external stress without wear mode transition. Then, at higher stresses (loads), fragments adhere more readily to the contact surface, which can lead to increased friction [38,39]. Again, the increase in friction heat generated with load leads to a significant temperature rise in the contact area, softening of HEA and an increase in wear rate, as can be expected [39]. The influence of frictional heat can be evaluated by parameter *Q_A_*, which represents the frictional heating power per unit time and per unit area, and can be calculated as follows [39,40]:

(6) $$Q_{A}=\frac{\mu PV}{A_{s}}$$

where *μ* and *P* are COF and normal load, respectively, whose product is equal to frictional force, *V* is the sliding speed and *A_S_* is the contact area of the worn surface. This means that when the COF and the contact area are fixed, greater loads and sliding speeds both contribute to increased frictional heat, and more pronounced heating effects and softening can be expected over time. In addition, higher surface temperatures can be achieved when heat generation and dissipation are balanced at higher loads and sliding speeds.

It is of great significance to understand the wear pattern of the nanostructured oxide layer and reveal its formation process [41,42,43,44,45,46]. According to the combination of abrasive wear, fatigue wear and oxidation wear, the wear process can be composed of four stages: (i) tight plastic deformation in the sliding process results in the surface damage of the HEA disk and the corresponding alumina ball, forming debris; (ii) the repeated grinding of surface debris leads to refinement and oxidation of hard debris in the air, and then cold welding or scratching the surface, leaving furrows and (iii) the brittle oxide layer gradually promotes crack propagation and fracture through stratification, forming pits and exposing unoxidized surfaces. Wear continues in this way, making the surface roughness uneven, which can be responsible for the high COF.

Eventually, the sources of good wear resistance are discussed specifically. The wear surface forms a dense oxide layer, avoiding the direct contact between HEA and the grinding ball, but the existence of the oxide layer is limited to the local area of the worn surface. The local presence of the oxide layer indicates that the oxide layer is unstable when it reaches a certain thickness, leading to the cracking and subsequent fracture of the oxide layer [43,44,45,46]. The evolution of the subsurface microstructure of AlCrFeNiV HEA helps to optimize its excellent wear resistance. The fine structure of HEA provides high-density grain boundaries and phase boundaries, which can act as a barrier to dislocation movement and deformation transfer (Figure 4). It is noted that the interaction between dislocation and hard phase (mainly BCC2) can improve the work hardening capability of the matrix. In addition, it is worth noting that the tight binding between the BCC1 and the BCC2 inhibits the formation of interfacial cracks, thus keeping the alloy stable during wear because the stress is concentrated at the interface of dislocation accumulation. In conclusion, the block fine-bearing particles with high hardness and mechanical stability are the key to the excellent wear resistance of HEA at present.

## 4. Conclusions

In summary, a new equiatomic AlCrFeNiV alloy with dual-phase BCC was obtained through the combination of high-energy ball milling and a spark plasma sintering process. This strategy can be beneficial in the development of new materials that can be applied in cutting tools.

The microstructure, microhardness and tribological properties were investigated in detail. The results are thus presented:

(1) Microhardness reached 1076 ± 15 HV0.5, leading to a proper wear resistance. In addition, the wear behavior of AlCrFeNiV against Al2O3 ball under dry wear and lubrication conditions has been investigated. Compared with dry friction, the COF of AlCrFeNiV HEA with lubricant decreased significantly, whereas the decrease in the wear rate was not meditative.
(2) The structure characterization indicates that two different kinds of body-centered cubic structures with different lattice parameters were formed in the AlCrFeNiV alloy, which is responsible for ultrahigh hardness.
(3) The wear mechanism was found to be abrasive wear and oxidation wear by analyzing the worn surface.

## Figures and Tables

**Table 1:** The relative parameters to predict the structure of AlCrFeNiV.

Parameter	Formula	Value
Δ*S_mix_*	$\Delta S_{mix}=-R\sum_{i=1}^{n}x_{i}\ln x_{i}$	13.38 KJ·mol^−1^
Δ*H_mix_*	$\Delta H_{mix}=\sum_{i=1,j=1}^{n}4\Delta H_{mix}C_{i}C_{j}$	−15.36 KJ·mol^−1^
*δ*	$\delta =\sqrt{\sum_{i=1}^{n}C_{i}(1-r_{i})}/(\sum_{i=1}^{n}C_{i}r_{i}{)}^{2}$	5.51%
*VEC*	$VEC=\sum C_{i}{\left(VEC\right)}_{i}$	6.40
χ	$\Delta \chi =\sqrt{\sum_{i=1}^{n}\chi_{i}{\left(\chi_{i}-\sum_{i=1}^{n}x_{i}\chi_{i}\right)}^{2}}$	12.78%

## Data Availability

The data that support the finding of this study are available from the corresponding author upon reasonable request.
